# Recent Insights in Islet Amyloid Polypeptide-Induced Membrane Disruption and Its Role in *β*-Cell Death in Type 2 Diabetes Mellitus

**DOI:** 10.1155/2008/421287

**Published:** 2008-05-06

**Authors:** Lucie Khemtémourian, J. Antoinette Killian, Jo W. M. Höppener, Maarten F. M. Engel

**Affiliations:** ^1^Department of Metabolic and Endocrine Diseases, Division of Biomedical Genetics, University Medical Center Utrecht, P.O. Box 85090, 3508 AB Utrecht, The Netherlands; ^2^Research group Chemical Biology and Organic Chemistry, Bijvoet Institute and Institute of Biomembranes, Utrecht University, Padualaan 8, 3584 CH Utrecht, The Netherlands

## Abstract

The presence of fibrillar protein deposits (amyloid) of human islet amyloid polypeptide (hIAPP) in the pancreatic islets of Langerhans is thought to be related to death of the insulin-producing islet *β*-cells in type 2 diabetes mellitus (DM2). The mechanism of hIAPP-induced *β*-cell death is not understood. However, there is growing evidence that hIAPP-induced disruption of *β*-cell membranes is the cause of hIAPP cytotoxicity. Amyloid cytotoxicity by membrane damage has not only been suggested for hIAPP, but also for peptides and proteins related to other misfolding diseases, like Alzheimer's disease, Parkinson's disease, and prion diseases. Here we review the interaction of hIAPP with membranes, and discuss recent progress in the field, with a focus on hIAPP structure and on the proposed mechanisms of hIAPP-induced membrane damage in relation to *β*-cell death in DM2.

## 1. INTRODUCTION

Long before discovery of the primary structure of the main component of amyloid in the islets of Langerhans, detailed ultra structural investigations had revealed that islet amyloid was often in contact with *β*-cell membranes [1]. In fact, it was found that amyloid fibrils were oriented perpendicular to the membrane of islet *β*-cells, with some fibril bundles sticking into membrane invaginations [1]. In 1987, the main component of islet amyloid was identified as a 37-amino acid residue peptide called islet amyloid polypeptide (IAPP) or amylin [2, 3]. Since then, the presence of IAPP amyloid at the *β*-cell membrane, and the concomitant morphological changes of these membranes, has been reported frequently [4–9]. These reports have contributed to the current hypothesis that the interaction between IAPP and cellular membranes could be a cause of IAPP cytotoxicity and *β*-cell death in DM2. Before reviewing IAPP-membrane interactions, we will briefly discuss the present knowledge on IAPP fibril formation.

## 2. IAPP FIBRIL FORMATION

### 2.1. From monomer to fibril

**Figure 1:**
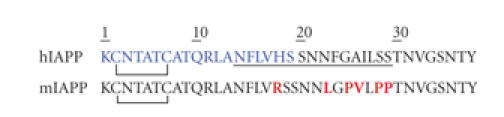
Comparison of the amino acid sequences of human IAPP (hIAPP) and mouse IAPP (mIAPP). Mouse IAPP differs from the human peptide by six residues (in red). The rectangle shows the N-terminal region that is thought to be important for membrane interactions. The amino acid region suggested to be important for fibril formation is represented in the underlined.

The amino acid sequence of IAPP varies slightly from organism to organism [10]. For instance, six residues are different between human IAPP (hIAPP) and mouse IAPP (mIAPP) (see Figure 1). Importantly, the latter does not aggregate into amyloid fibrils, and amyloid is generally not observed in the pancreas of wild-type mice. Nevertheless, transgenic mouse models that express human IAPP develop fibrillar deposits and exhibit signs of diabetes [11].

**Figure 2:**
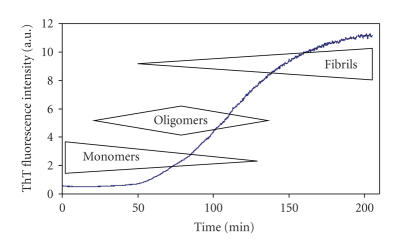
Typical shape of the kinetics of hIAPP fibril formation, characterized by a lag phase and a sigmoidal transition. The approximate aggregation state of IAPP is indicated at the various time points. Fibril formation was induced by adding, at time 0, a monomeric stock solution of hIAPP in DMSO to buffer containing Thioflavin T.

The in vitro aggregation and fibril formation of hIAPP have been studied extensively in the last years [12–22]. In most of these studies, hIAPP aggregation is initiated by dilution of, usually synthetic, monomeric hIAPP into a physiological buffer. This results in the “spontaneous” aggregation of hIAPP monomers into amyloid fibrils, as can be observed, for example, by electron microscopy. The in vitro aggregation of hIAPP is typically completed in a few hours, depending amongst others on peptide concentration and presence of lipids [18]. This is significantly faster than the aggregation of most other amyloidogenic peptides. Fibril formation of IAPP, as well as of some other amyloidogenic peptides, generally occurs via a nucleation dependent aggregation process [18, 23]. This means that the formation of a nucleus, usually a slow step, is required for initiation of the growth of stable fibrils. The nucleus is an ordered oligomeric hIAPP species that can serve as a template for fibrillar hIAPP. The kinetics of hIAPP fibril growth can be monitored in time by the commonly used method of specific binding of the fluorescent molecule Thioflavin T (ThT) to amyloid fibrils [24]. A kinetic trace of hIAPP fibril growth shows a lag phase and a sigmoidal transition that are typical for fibril growth of amyloidogenic proteins and peptides (see Figure 2) [23]. After dilution of initially monomeric hIAPP in buffer, the thermodynamically unfavourable process of nucleation occurs, although the initial horizontal baseline of the ThT curve indicates that no fibrils are formed in the beginning (lag phase). The sigmoidal increase in the ThT curve indicates propagation of fibril growth with consumption of monomer. Next to the monomeric and fibrillar states of hIAPP, several intermediate (oligomeric) states have been observed, as will be discussed later. Elongation of fibrils proceeds via addition of monomers or oligomers to both fibril ends.

### 2.2. Three-dimensional molecular structure of hIAPP

The three-dimensional structure of amyloid fibrils, and lately also the structure of monomers and oligomers, has been the subject of research into the molecular background of amyloid diseases. However, only little structural information is available for the IAPP monomer, oligomer, and fibril. In 1992, the first, limited information of the three-dimensional structure of soluble hIAPP was obtained [25]. It was shown that hIAPP exhibits a random coil structure with small components of *α*-helical and *β*-sheet conformations. Recent studies confirmed that soluble hIAPP has mainly unordered backbone structure [26–28]. In contrast, hIAPP dissolved in the organic solvent trifluoroethanol (TFE, a membrane mimicking solvent) predominantly adopts an *α*-helical conformation [25]. Our observations have indicated that hIAPP dissolved in TFE initially adopts *α*-helical structure, before transforming into *β*-sheet structure (unpublished results). These observations suggest that hIAPP could also adopt *α*-helical structure in a membrane environment.

hIAPP oligomers or aggregates ranging from dimers up to 6000 molecules have been reported by several research groups [7, 29–31]. These oligomers appear to represent intermediates on the path to fibril formation. There are recent indications that hIAPP oligomers, in presence of membranes, exhibit *α*-helical structure [28]. This is surprising since it would seem thermodynamically unfavourable for a monomer with random coil structure to first adopt *α*-helical structure before changing into *β*-sheet rich fibrillar structure. Aggregation intermediates have been observed for many types of amyloid proteins, such as *α*-synuclein and A*β* [32, 33]. Glabe and coworkers have produced a conformation-dependent antibody that is specific for soluble oligomers and does not recognize natively folded proteins, monomer, or fibrils [34]. They showed that this antibody recognizes soluble oligomers from a wide variety of amyloid-forming peptides and proteins such as hIAPP, Prion 106–126, human insulin, A*β* peptide, and polyglutamine, which suggests that these oligomers might have a common structure.

The three-dimensional structure of hIAPP fibrils has been studied by various high-resolution techniques, like electron microscopy, X-ray diffraction, electron diffraction, and electron paramagnetic resonance [26, 35–38]. These studies clearly reveal that hIAPP fibrils contain a significant amount of well-ordered cross-*β* structure, typical of amyloid fibrils. During fibril formation, hIAPP undergoes a conformational change from random coil to a mixture of *β*-sheet and *α*-helical structure [26]. These results are consistent with the work of Kayed [15], who also measured a random coil to *β*-sheet transition for hIAPP fibril formation. hIAPP fibrils are polymorphic, ranging from thin protofilaments with a diameter of about 5 nm to thicker fibrils with diameter of up to 15 nm that appear to be rope-like bundles of protofilaments. The predominant type of fibril contains three protofilaments in a left-handed coil with a pitch of 25–50 nm.

### 2.3. Which amino acid residues are important for hIAPP fibril formation?

Structural studies have shown that amino acid residues 20–29 of hIAPP are crucial for amyloid formation [12]. A proline scan of this decamer (hIAPP20–29) has demonstrated that substitution of a single proline at either position 22, 24 or at positions 26–28 leads to a drastic reduction of amyloid formation [39]. Note that three of the six differences between hIAPP and the nonamyloidogenic mIAPP involve a proline, a residue that is predicted to disrupt ordered structure, like the *β*-sheet structure in amyloid fibrils.

Currently, research groups are developing molecules in an attempt to reduce hIAPP-induced *β*-cell death by inhibiting hIAPP fibril formation. Some of these “inhibitors” are based on synthetically modified hIAPP peptides or hIAPP fragments that are not able to form fibrils themselves, but are suggested to bind to, and to stop the elongation of growing hIAPP fibrils [40–42]. A recent study indicated that a single amino acid substitution in hIAPP, where Ile on position 26 is replaced by Pro (I26P), yields a potent fibrillization inhibitor [43].

Although residues 20–29 play an important role in hIAPP fibril formation, these may not be the only residues involved. It has been hypothesized that aromatic-aromatic interactions are also important in hIAPP fibril formation [44]. Human IAPP contains three aromatic residues at positions 15, 23, and 37 (see Figure 1). The aromatic-aromatic and aromatic-hydrophobic interactions in amyloid formation were studied using a hIAPP triple mutant [45]. The triple mutant F15L/F23L/Y37L, lacking aromatic residues, still forms amyloid fibrils in vitro, indicating that the aromatic residues are not essential in hIAPP fibril formation. However, the substitution decreases the rate of fibril formation and alters the tendency of fibrils to aggregate. Some studies demonstrate that the amino acid region from residues 11 to 20 is also important for hIAPP fibril formation [46, 47]. A recent study shows that the hIAPP fragment consisting of residues 14–20 can form amyloid fibrils [38].

hIAPP contains a single histidine at position 18 (see Figure 1), which is the only residue in this peptide that has a charge that depends on pH in a physiological pH range. Consequently, fibril formation of hIAPP could depend on the pH. A recent study showed that hIAPP fibril formation is faster at a lower pH (4.0) than at a higher pH (8.8) [48]. This could be important in a physiological context since in the *β*-cell granules of the pancreas, where hIAPP is stored, the pH is 5.5, but when hIAPP is released into the extracellular compartment, it experiences a pH of 7.4 [49].

Another characteristic of hIAPP is the intramolecular disulfide bond between cysteines residues 2 and 7 (see Figure 1). The disulfide does not contribute to the amyloid fiber core structure; however it somehow must play a central role in the assembly mechanism, since loss of the disulfide significantly reduces fibril formation [20].

## 3. hIAPP AGGREGATION AND FIBRIL FORMATION IN THE PRESENCE OF MEMBRANES

### 3.1. Membrane phospholipids catalyse hIAPP fibril formation

It has been observed that phospholipid membranes promote the aggregation of hIAPP [28, 50, 51]. In the presence of phospholipids, the kinetic profile of hIAPP fibril growth is characterized by a reduction in the lag time resulting in earlier fibril formation [50]. Cellular membranes could accelerate hIAPP fibril formation by enhancing nucleation. The lipid composition may play an important role in this process, since it has been demonstrated that hIAPP aggregation is accelerated in the presence of membranes that contain negatively charged lipids such as 1,2-dioleoyl-*sn*-glycero-3-phospho-L-serine (DOPS) or 1,2-Dioleoyl-sn-Glycero-3-[Phospho-rac-(1-glycerol)](DOPG) [27, 50, 51]. In the presence of such membranes, hIAPP fibril formation occurs within a few minutes as opposed to a few hours in the absence of membranes [27, 50]. A membrane-induced change in the conformation in hIAPP could possibly result in formation and/or stabilization of a nucleus, which could in turn result in acceleration of hIAPP fibril formation. Hence, elucidation of the conformation of hIAPP in interaction with the membrane is an important issue. Knowledge of this conformation would give valuable insights into the mechanism of membrane damage and would aid in developing new drugs and/or finding new targets for the treatment of DM2.

### 3.2. Insight in the conformation of membrane-interacting hIAPP

Recently, studies have been performed to determine the conformation of hIAPP that interacts with model membranes, that is, large unilamellar vesicles (LUVs) [27, 28, 52]. In these studies, the LUVs are composed of a combination of a neutral phospholipid, for instance, 1,2-dioleoyl-*sn*-glycero-3-phosphocholine (DOPC), and a negatively charged phospholipid, for instance, 1,2-dioleoyl-*sn*-glycero-3-phospho-L-serine (DOPS). In the presence of LUVs, hIAPP initially displays *α*-helical structure [27], corresponding with the structure of hIAPP in the membrane-mimicking solvent TFE [25]. However, after 40 minutes incubation with LUVs, the conformation of hIAPP changes to predominantly *β*-sheet conformation, characteristic of fibril formation [27]. Recently, the structure of hIAPP in membrane bilayers was studied using microscopy techniques [53]. It was found that hIAPP forms pores that are composed of five subunits, in which each subunit is suggested to represent an hIAPP monomer. This hIAPP morphology was connected to channel-like behavior in planar bilayers, indicating that these oligomeric hIAPP pores could incorporate in membranes and change their barrier properties. Unfortunately, high-resolution structural information of hIAPP in a membrane environment is still lacking, mainly because of the instability of the membrane-interacting hIAPP aggregates. Preliminary results of our group indicate that hIAPP fibrils grown in the presence of phospholipids have the same characteristic structure as fibrils formed in the absence of lipids.

### 3.3. Which residues are important in the interaction with membrane?

It is likely that the presence of membranes causes additional residues in IAPP to be involved in fibril formation, as compared to the situation without membranes. Several residues that are important for hIAPP-membrane interactions can be identified. It can be anticipated that the positively charged residues, which are all located at the N-terminal part of hIAPP at positions 1, 11, and 18 (see Figure 1), will be important in the interaction of hIAPP with negatively charged phospholipid membranes. Indeed, there are indications that hIAPP molecules cluster at the membrane surface, prior to fibrillogenesis, with their N-termini oriented towards the membrane [50]. More recently, it was shown that an N-terminal hIAPP fragment (hIAPP1–19) has a significantly higher ability to insert in phospholipid monolayers than a fragment from the central, amyloidogenic region of hIAPP (hIAPP20–29) [54]. These findings suggest that the N-terminal part of hIAPP, whilst not significantly involved in hIAPP fibril growth, is important in light of hIAPP-membrane interactions.

## 4. MECHANISM OF CYTOTOXICITY

In 1993, work on the amyloidogenic Alzheimer's related peptide Abeta had indicated that an amyloidogenic protein can form ion-selective membrane channels, providing a first hypothesis for the mechanism of amyloid (neuro)toxicity [55, 56]. The observations that IAPP fibrils are located at the cellular membrane in the Islets of Langerhans and that this is accompanied by alterations in membrane morphology [1, 4–9] made researchers hypothesize that the membrane might be the target of cytotoxic IAPP and that this could cause death of the insulin producing *β*-cells, similar to Abeta neurotoxicity in Alzheimer's disease. The first experimental evidence that indeed hIAPP can cause membrane disruption came from work by the Kagan group [57]. It was found from experiments with planar lipid bilayers that synthetic hIAPP forms ion-permeable channels “pores” in the membrane, whereas the nonamyloidogenic mouse IAPP does not form channels. Mature hIAPP fibrils were found to be less cytotoxic; moreover, they did not cause significant membrane disruption in comparison to oligomeric hIAPP [58, 59]. Still, the exact mechanism of hIAPP-induced membrane disruption is far from understood, and various mechanisms have been hypothesized during the last 10 years [7, 29, 31, 34, 50, 51, 54, 57, 60–63]. It is, for example, unclear what the exact nature of the hIAPP species that interacts with or even disrupts membranes is. The main hypothesis, based on in vitro evidence, suggests a major role for a specific prefibrillar hIAPP aggregate, commonly known as hIAPP oligomer, as the membrane-disrupting species [7, 29, 31, 34, 57, 59, 60, 64]. The various suggested mechanisms for hIAPP-induced membrane disruption will be discussed below.

### 4.1. hIAPP oligomers cause membrane damage and are cytotoxic

Recently, it has been suggested that prefibrillar aggregates (or oligomers), formed early during aggregation and not mature amyloid fibrils are the cytotoxic species in protein misfolding diseases [65]. Considering amyloid cytotoxicity in DM2, the prevailing view is that IAPP-induced membrane damage, and concomitant *β*-cell death, is caused by cytotoxic hIAPP oligomers [7, 28, 29, 31, 53, 57, 60, 64]. There are indications that these oligomers form ion channels [53, 57], as has been suggested for other amyloidogenic proteins [55, 66]. Other studies indicate that hIAPP oligomer-induced membrane damage is not specific for ions [31] but results in membrane leakage of molecules with a size of up to 600 Da (Calcein), indicating a general membrane disruption mechanism by hIAPP oligomers [28, 51, 59, 63, 67].

Small hIAPP aggregates have been shown to be cytotoxic in cell cultures, and these aggregates were also able to destabilize model membranes [7]. Similarly, oligomeric hIAPP was found to form membrane pores, allowing molecules with the size of a calcium ion to pass. These pores disappeared, and membrane damage decreased, when hIAPP fibrils grew and oligomers were consumed [29, 60]. Electron microscopy analysis showed that hIAPP formed spherical shapes with a diameter of 3 to 20 nm, consistent with the presence of hIAPP oligomers [31, 60]. In a test tube, oligomeric hIAPP can be prepared under specific experimental conditions. Addition of such preparations to human neuroblastoma cells that were loaded with fluorescent dye resulted in the cellular leakage of this dye [59]. This indicates that hIAPP oligomers, when applied to the outside of cells, are cytotoxic via a general membrane destabilizing effect and not via a specific ion pore. The monomeric and fibrillar form of hIAPP clearly did not have this effect. Later it was also shown that when applied from the inside of cells, using cells that overexpress hIAPP, the hIAPP oligomers are also able to perform their cytotoxic action [64]. Recently, it has been suggested that ER and mitochondrial membranes might be the target of cytotoxic hIAPP, resulting in ER stress and *β*-cell apoptosis [68]. Moreover, intracellular hIAPP oligomers were indirectly demonstrated in the pancreatic *β*-cells of hIAPP-transgenic mice using an oligomer-specific antibody [69]. The latter study also showed that oligomer-specific antibodies could not prevent hIAPP-induced *β*-cell death, indicating that toxic events might occur inside the cell.

**Figure 3:**
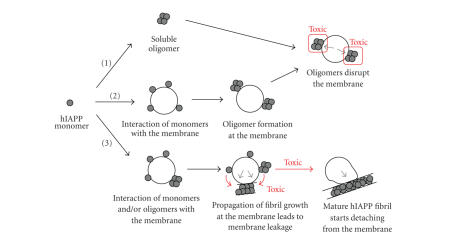
Simplified schematic representation of the different models of hIAPP-membrane interaction in relation to membrane damage and hIAPP cytotoxicity. The red rectangles show the toxic species and the red arrows show the toxic processes according to different hypotheses. The black circle represents a phospholipid membrane (vesicle), the grey circles represent hIAPP monomers, and clusters of 4 or more circles represent hIAPP oligomers and hIAPP fibrils, respectively. Membrane damage is schematically indicated by the grey arrows. Model (1) includes two steps: (i) formation of soluble hIAPP oligomers, (ii) interaction of the toxic oligomers with the membrane leading to membrane damage. Model (2) includes three steps: (i) binding of monomeric, random coil hIAPP to the membrane and folding to *α*-helix, (ii) oligomer formation of membrane-bound hIAPP, and (iii) interaction of the toxic hIAPP oligomer with the membrane leading to membrane damage. Model (3) includes 3 steps: (i) interaction of monomeric and possibly oligomeric hIAPP to the membrane, (ii) growth of hIAPP fibrils at the membrane (red arrows) leading to a forced change in membrane morphology and concomitant membrane disruption, and (iii) detachment of mature fibrils from distorted membrane.

The exact mechanism of membrane disruption by hIAPP oligomers is not known. Some groups show that preassembled hIAPP oligomers disrupt membranes [31, 60], whereas others suggest that hIAPP monomers first interact with the membrane and only then form oligomeric hIAPP with membrane disrupting capacity [28]. These two models of membrane damage by hIAPP oligomers have been schematically depicted in Figure 3.

In conclusion, many observations indicate that hIAPP oligomers are a likely candidate for inducing cell death. In contrast, hIAPP fibrils are found not to damage membranes and could in fact be the result of a physiological mechanism in which toxic oligomer species are disposed of in a nontoxic, fibrillar form.

### 4.2. Membrane damage by fibril growth at the membrane

In addition to the hypothesis that oligomers are the toxic species, recent reports suggest also other mechanisms for hIAPP cytotoxicity. One such hypothesis is that membrane damage is not caused by a specific hIAPP species, such as an oligomer, but by the process of fibril growth at the cellular membrane. There are several recent indications that growth of hIAPP fibrils at the membrane can cause membrane damage. In this model, the initial steps of the interaction of hIAPP with membranes are adsorption, followed by insertion of hIAPP into the membrane, either as monomer or as oligomer (see Figure 3). The interaction of monomeric hIAPP with membranes is likely as monomeric hIAPP has a strong tendency to insert in phospholipid monolayers [52, 54]. In the next step, interactions of membrane-located hIAPP species with each other, or with hIAPP species in solution, lead to growth of fibrils at the membrane (model 3 in Figure 3). The mechanism of membrane damage could entail growth of a rigid hIAPP fibril on a flexible phospholipid bilayer, which would result in a forced change in membrane curvature. This change in membrane curvature leads to deformation of the shape of the membrane. Interestingly, disruption, blebbing and vesicle budding of cell membranes in the presence of synthetic [5, 7, 9] and cell-derived hIAPP [6, 8, 70] have been noticed in many studies. Our recent results indicate that the kinetics of membrane damage is very similar to the kinetics of fibril formation (see Figure 2). Both processes, fibril formation and membrane damage, were characterized by the presence of a lag phase and a strong enhancing effect on the kinetics upon the addition of seeds [71]. In case of the Alzheimer's disease-related Abeta peptide, it has been suggested recently that not a particular species but ongoing amyloid fibrillogenesis is responsible for membrane damage [72]. Together, these notions suggest that a cytotoxic mechanism based on fibril growth at the membrane could represent a common mechanism for amyloid-induced cell death. Finally, another factor that could contribute to membrane damage by fibril growth is uptake of membrane lipids in amyloid, a phenomenon that has been observed, both in vitro [51, 73, 74] and in vivo [75].

## 5. INITIATION OF HARMFUL IAPP-MEMBRANE INTERACTIONS IN DM2

Since the combination of hIAPP and membranes in nondiabetic people does not normally result in *β*-cell death; certain DM2-related conditions should exist that initiate hIAPP-induced membrane damage. An increase in the level of hIAPP, which is coproduced and cosecreted with insulin, in a state of insulin resistance, could initiate hIAPP fibril formation. More specific, an altered ratio of insulin to hIAPP, as observed in diabetic patients [16], could lead to a decrease of the inhibitory effect of insulin on hIAPP amyloid fibril formation. This inhibitory effect of insulin on hIAPP fibril formation has been observed in vitro [76–78]. On the other hand, a changing lipid composition of the *β*-cells, in particular an increase in negatively charged lipids as inferred from studies with mouse and rat models for DM2 [79], could also trigger an increase in hIAPP-membrane interactions. In vitro studies show that negatively charged lipids increase the rate of hIAPP fibril formation [27, 50] and also enhance hIAPP-induced membrane damage [28, 51]. The membrane itself could promote hIAPP growth by increasing the local concentration of (membrane bound) hIAPP and/or by promoting a specific orientation or conformation of the peptide that makes hIAPP molecules more susceptible to aggregation into oligomers or fibrils. Recent research shows that not only phospholipid bilayers, but also a polyanion like heparin [80] or a dichloromethane/water interface [22] can induce nucleation and aggregation of hIAPP. These results indicate that charge and a hydrophobic/hydrophilic interface (both present in biological membranes) are important factors that promote hIAPP fibril formation.

## 6. FUTURE PERSPECTIVES AND CHALLENGES

During the last years, the understanding of hIAPP-membrane interactions has significantly increased. We have now important indications that oligomeric hIAPP, in contrast to fibrillar hIAPP, is the main species involved in membrane damage and is a likely candidate to cause *β*-cell death in DM2. However, in a cellular environment, such toxic oligomers have not (yet) been directly demonstrated. More insight is required into the question whether hIAPP oligomers are inherently cytotoxic and persist as toxic oligomer after their cytotoxic action, or whether they are transient participants in the process of fibril growth at the membrane. A major challenge is to elucidate the mechanism by which hIAPP induces membrane damage and cytotoxicity. This knowledge would be essential to develop new strategies to battle hIAPP-induced *β*-cell death in DM2. Determination of the three-dimensional structure of membrane disrupting hIAPP would be an important contribution in elucidation of the cytotoxic mechanism. Moreover, the importance of hIAPP-membrane interactions, discussed here, indicates that inhibition or alteration of hIAPP-membrane interactions might be an alternative strategy to reduce amyloid cytotoxicity and to prevent *β*-cell death in DM2, in addition to the “traditional strategy” to reduce amyloid by the development of molecules that inhibit amyloid fibril formation.
